# An Industrial Digitalization Platform for Condition Monitoring and Predictive Maintenance of Pumping Equipment

**DOI:** 10.3390/s19173781

**Published:** 2019-08-31

**Authors:** Michael Short, John Twiddle

**Affiliations:** 1 School of Science, Engineering and Design, Teesside University, Middlesbrough TS1 3BA, UK; 2 Scottish & Southern Energy Ltd., Knottingley, West Yorkshire WF11 8SQ, UK

**Keywords:** industrial digitalization, industry 4.0, sliding mode observers, soft sensors, edge computing, condition monitoring, predictive maintenance, field testing, IoT

## Abstract

This paper is concerned with the implementation and field-testing of an edge device for real-time condition monitoring and fault detection for large-scale rotating equipment in the UK water industry. The edge device implements a local digital twin, processing information from low-cost transducers mounted on the equipment in real-time. Condition monitoring is achieved with sliding-mode observers employed as soft sensors to estimate critical internal pump parameters to help detect equipment wear before damage occurs. The paper describes the implementation of the edge system on a prototype microcontroller-based embedded platform, which supports the Modbus protocol; IP/GSM communication gateways provide remote connectivity to the network core, allowing further detailed analytics for predictive maintenance to take place. The paper first describes validation testing of the edge device using Hardware-In-The-Loop techniques, followed by trials on large-scale pumping equipment in the field. The paper concludes that the proposed system potentially delivers a flexible and low-cost industrial digitalization platform for condition monitoring and predictive maintenance applications in the water industry.

## 1. Introduction

With improved connectivity and dramatically increased access to low-cost computational power, many industries are currently on the verge of a second digital revolution known as ‘Industry 4.0′ [1]. Of the many advantages that can potentially be leveraged by Industry 4.0, the promise of increased integration of the real-time control, monitoring, and operational aspects of equipment in the process industries is one of the most attractive [1,2]. It has previously been suggested that one of the so-called grand challenges which can be addressed by digitization is the notion of the ‘sustainable, 100% available plant’ [1]. The 100% availability notion captures the vision that in the future there will be only ever be planned maintenance stops; while clearly never achievable in practice, the right balance between maintenance cost and risk must clearly be aimed for. At the heart of this aspect of industrial digitalization the topics of real-time Condition Monitoring (CM) and Predictive Maintenance (PM) can be found [1,2]. As with other Internet-of-Things and Industry 4.0 applications, CM/PM applications can potentially generate large volumes of high-velocity real-time data; therefore, it is beneficial to run local processing of this data at the edge of the network (on an ‘edge device’), to reduce the frequency and real-time requirements of communication to core devices [3]. In addition, since it is essential to have adequate fault indices that could serve as indicators in order to predict early failures and create an effective PM schedule [2], in an Industry 4.0 context an appropriate approach would seem to be to distribute the tasks of CM/indices generation and PM analytics between the network edges and Core.

To this end, this paper is concerned with the development, validation and field-testing of a real-time CM/PM system for deployment upon large-scale pumping equipment in the water industry in an Industry 4.0 context. The CM and fault detection is achieved with an edge device implementing sliding-mode observers as soft sensors, which are designed to monitor equipment locally in a water pumping station. The role of the observers is to detect equipment wear - from mechanical stresses, or the ingress of coolant or other particulates into coupling bearing support housing - before further damage occurs to the equipment. It achieves this by monitoring several low-cost transducer signals from the equipment, and building a digital twin of the equipment being monitored; including the estimation of two critical internal parameters of the rotating equipment (bearing friction factor and coolant flow) which are impossible or difficult/expensive to directly measure. The estimated values (in conjunction with other instrumented measurements) are used as fault diagnosis indices. The edge device also performs data sampling and filtering and provides outputs for fault detection in the form of local status indicators and alarms. A Modbus RTU server is implemented on the device, and wider connectivity to the network core is provided through an additional Serial to Mobile Data (3G/4G) gateway. The core is able to pull preprocessed and summarized digital data related to local real-time parameter measurements and estimations at a lower velocity through this gateway, where further analytics related to PM are applied; this provides an architecture in keeping with the decentralized, hierarchical structure of the digitalization revolution and IoT [1,2,3]. The focus of this paper is principally upon the implementation and testing of the edge device and soft sensors for CM.

Given the relative importance of the ‘100% available plant’ aspirational target, the CM/PM application in question can be considered to be mission-critical as any ‘false negatives’ indicated by the system may result in avoidable permanent damage to equipment, and ‘false positives’ may cause unnecessary system downtime. Both unwanted outcomes may lead to large and unnecessary economic costs and can potentially interrupt a region’s critical water supply infrastructure. It is therefore of vital importance to ensure that adequate Verification and Validation (V&V) exercises and prototype field trials are carried out on any implementation of this edge device. This paper describes such a set of exercises, carried out on a prototype Commercial Off The Shelf (COTS) microcontroller-based embedded solution, which is to be deployed locally in a pump house; a potentially harsh industrial environment. 

Much previous work has been invested into developing accurate condition monitoring of rotating equipment in industrial applications; many focus on the use of vibration or acoustic analysis using real-time data processing techniques such as Wavelet transforms, Machine Learning approaches and Parameter Optimization (see, e.g., [2,4,5,6,7,8]). However, the specific focus of this paper is on sliding mode observer techniques, which have also been widely used for condition monitoring and fault diagnosis in recent years (see e.g., [9,10,11]). Two of their main advantages are that they exhibit fundamental robustness against certain kinds of parameter variations and disturbances, while also avoiding the need for high-speed signal processing techniques required for acoustic or vibration data; a specialized Sliding Mode Observer (SMO) for fault detection and isolation was developed by Edwards et al. to exploit these features [10]. Additional key benefits of this observer approach include good accuracy of operation, combined with comparatively lower-overheads than related techniques [9,10,11], making them a good candidate for real-time implementation in an Industry 4.0 context. Therefore, the principal goal of this paper is to help bridge the theory and practice gap by providing a detailed description and credible demonstration of an industrial implementation of a simple but effective edge device implementing this observer-based technique for fault detection of pumping equipment. Both the observer itself, and the techniques applied to implement the observer in real-time on a small, low-cost portable embedded system, have a fundamental theoretical grounding. Through laboratory-based validation using fault injection, combined with online field trials, it is demonstrated that the developed system seems a good candidate for wider deployment in industry to help achieve the goals of the second digital revolution.

The remainder of this paper is organized as follows. Section 2 describes the current application area in more depth, along with the motivation for using observers as soft sensors and their mode of operation. Section 3 describes the prototype hardware and software implementation of the edge device, along with a discussion of communication network integration. Section 4 discusses off-line validation testing of the device, while Section 5 is concerned with on-line testing (field trials). Section 6 concludes the paper.

## 2. Sliding Mode Observers as Soft Sensors

### 2.1. Sliding Mode Techniques

**Figure 1:**
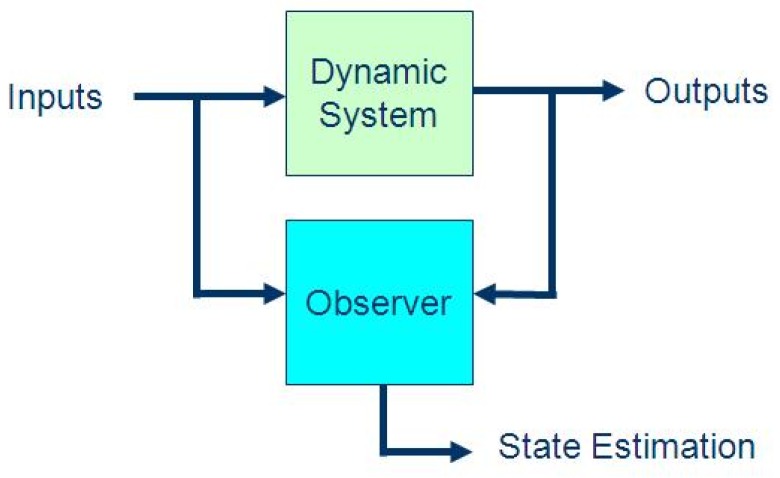
Basic operation of a sliding mode observer.

As shown in Figure 1, a sliding mode observer is a state observer that employs sliding mode techniques to give accurate estimates of a system’s internal state in the presence of uncertainty and parameter variations. The observer is designed to maintain the sliding motion, even in the presence of faults, and reconstructs fault signals as a function of the so-called equivalent output injection signal [9,10,11]. These estimates of the unmeasured fault signals can then be used as measures of equipment health, and employed to raise alarms or to schedule maintenance prior to critical failures occurring in the equipment being monitored. The design procedure for this model-based estimation approach is principally characterized by two components: (i) the design of an appropriate sliding surface where the system will demonstrate the desired dynamics and (ii) the selection of an injection signal that will force the system to reach and maintain its sliding motion [9,10,11]. 

### 2.2. Application in the Water Industry

**Figure 2:**
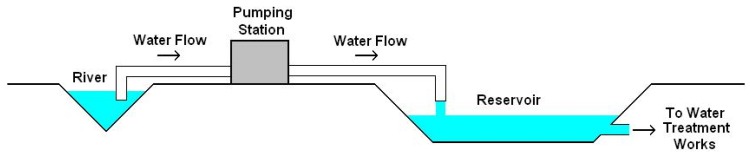
Overview of current application area.

Much of the industrial plant in the UK water industry consists of large-scale rotating machinery such as can be found in pumping stations. For example, in the first stage of a water treatment works a regulated supply of water (from a reservoir) is necessary. As precipitation is unpredictable, water can be drawn from a constant supply—such as a river—that is close to ground level to regulate the reservoir level, as shown in Figure 2. In such an application, a large amount of water may have to be pumped a considerable distance in order to regulate the mains water supply. The equipment in such stations is required to have a high level of availability, and is relatively expensive to repair if damage to couplings and shafts occurs. The current application is concerned with monitoring the health status of the main shaft support bearing in this kind of pumping equipment, to detect possible fault conditions such as increased wear, water ingress or coolant flow failure.

The shaft support bearings are often monitored using spectral analysis of vibration signals [5]. Increased temperature is also a common symptom of bearing wear or of a lubrication failure [11]. However, several factors such as ambient temperature, load or instantaneous rotational shaft speed can also cause natural variations in the measured bearing temperature; a simple limit or threshold approach to detection of overheating is not generally acceptable, as it needs to accommodate a wide range of ambient and operating conditions whilst minimizing the number of false alarms. The robustness of the sliding mode approach can take account of these environmental and operational factors, efficiently eliminating them from the fault detection process, and thus increasing the sensitivity of the diagnosis system reducing the number of false positives. Compared with spectral analysis, temperature monitoring using reliable but inexpensive transducers and sliding mode techniques offers many potential benefits, including lower overall costs, lower sampling rates, and less computationally intensive signal processing techniques [9,10,11]. A temperature monitoring approach also avoids the problem of background noise and vibration from other pumps interfering with the data. Additionally, the low sampling rates and computational intensity would seem to indicate that low-cost ADC’s and microcontroller units may be employed to realize the CM system in a simple edge device; this provides the motivation for their deployment in the current work.

### 2.3. Technical Details

**Figure 3:**
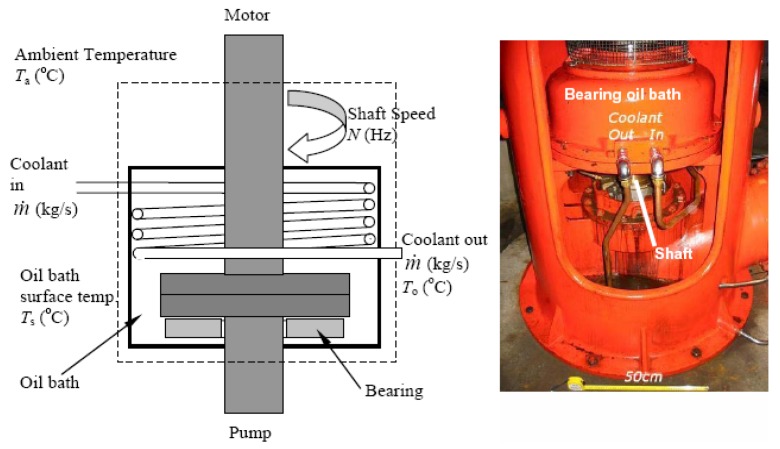
Detail of the shaft coupling.

Previous work by Kitsos et al. [11] has shown that a multiple observer approach—with the observers designed as virtual or ‘soft’ sensors—to monitor coolant flow and bearing wear respectively provides a highly efficient solution. In general terms, the observers are designed to detect early predictors of bearing overheating due to either: (i) loss or reduction of coolant flow to the bearing, e.g., if the water sump runs dry or (ii) increased friction; e.g., due to wear and tear, reduction in oil level, ingress of particles or water into bearing itself. A schematic and picture of the bearing in the current application is shown in Figure 3.

The observers themselves are based upon two simplified heat transfer models of the arrangement shown in Figure 3. Equation (1) captures the coolant heat transfer dynamics, while Equation (2) captures the bearing heat transfer dynamics:

(1) $$\begin{array}{ccc} {\dot{Q}}_{Coolant} & = & {\dot{Q}}_{FromBearing}-{\dot{Q}}_{CoolantOut}\\ m_{C}C_{C}{\dot{T}}_{o} & = & h_{c}A_{c}(T_{B}-T_{o})-{\dot{m}}_{c}C_{c}(T_{o}-T_{i})\\ {\dot{T}}_{o} & = & b_{1}(T_{B}-T_{o})-{\dot{m}}_{c}b_{2}(T_{o}-T_{i}) \end{array}$$

(2) $$\begin{array}{ccc} {\dot{Q}}_{Bearing} & = & {\dot{Q}}_{Friction}-{\dot{Q}}_{CoolantOut}-{\dot{Q}}_{Atmosphere}\\ m_{B}C_{B}{\dot{T}}_{B} & = & \mu F_{f}N-{\dot{m}}_{c}C_{c}(T_{o}-T_{i})-h_{B}A_{B}(T_{B}-T_{a})\\ {\dot{T}}_{B} & = & a_{1}N-a_{2}(T_{o}-T_{i})-a_{3}(T_{B}-T_{a}) \end{array}$$

where *T_i_* and *T_o_* are the coolant inlet/outlet temperatures (°C) respectively, *T_B_* is the measured bearing temperature (°C), *T_a_* is the ambient temperature (°C) and scalars *a_i_* and *b_i_* are appropriate model coefficients employed to simplify the expressions. The coolant mass flow rate is denoted $\dot{m}$ (kg/s). The corresponding sets of equations defining the coolant and bearing observers are given below in Equations (3) and (4) respectively:

(3) $$\begin{array}{ccc} {\hat{\dot{T}}}_{o} & = & b_{1}(T_{B}-{\hat{T}}_{o})-\tilde{\dot{m}}b_{2}({\hat{T}}_{o}-T_{i})-\nu_{o}\\ \varepsilon_{o} & = & {\hat{T}}_{o}-T_{o}\\ \nu_{o} & = & K_{o}\varepsilon_{o} \end{array}$$

(4) $$\begin{array}{ccc} {\hat{\dot{T}}}_{B} & = & {\tilde{a}}_{1}N-a_{2}(T_{o}-T_{i})-a_{3}({\hat{T}}_{B}-T_{a})+\nu_{B}\\ \varepsilon_{B} & = & {\hat{T}}_{b}-T_{b}\\ \nu_{B} & = & K_{B}\varepsilon_{B} \end{array}$$

where *ν_B_* and *ν_O_* are the ‘injection signals’ for the coolant outlet temperature and bearing observers respectively, and *ε_i_* are the model errors. Note that an accented variable denotes the observer model estimation. The observer is designed such that the state error signals are driven toward zero. When the state error is small, any changes in parameters $\dot{m}$ and *a*_1_ caused by loss or reduction in coolant flow or because of increased heating due to friction can be approximated using Equations (5) and (6) as follows:

(5) $$\Delta \dot{m}\approx \frac{\nu_{o}}{b_{2}(T_{o}-T_{i})}$$

(6) $$\Delta a_{1}\approx \frac{\nu_{B}}{N}$$

where *N* is the shaft speed (in Hz). The observer has been found to work well under purely simulation conditions, but to be of practical use in the field an implementation that is capable of functioning correctly in the relatively harsh environment of a pumping station is required. Remote connectivity to a control center for data analysis, logging, and predictive maintenance scheduling is also required. The hardware, software, and communication architectural design of a prototype edge device for this purpose is described in the next Section.

## 3. Prototype Edge Device

### 3.1. Hardware Architecture

**Figure 4:**
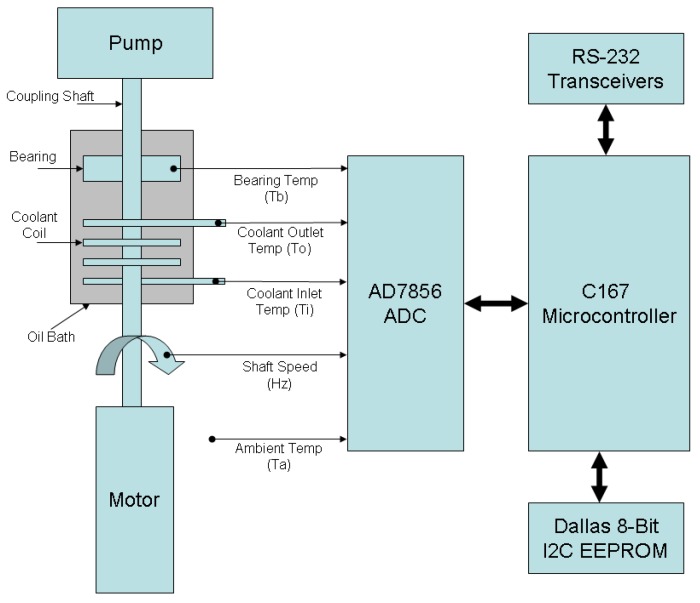
Main hardware elements employed in the system.

Several possibilities were considered for implementing the edge device in hardware, including the use of Raspberry Pi and Arduino-based devices. However, the microcontroller employed in this prototype was an Infineon© C167CS housed in a Phycore-167 SBC (Single-Board-Computer) unit. This unit was mounted on a development board purchased from Phytec©. This configuration was chosen due to the suitability of the device for deployment in harsh industrial environments (the C167 microcontroller was originally developed for automotive/in-vehicle applications), and the availability of a reliable/mature development toolchain along with proven static code analysis and execution time profiling tools. The host CPU features 4 KB of on-chip RAM (IRAM), the CPU and associated peripherals. The SBC consists of the host CPU, an oscillator/reset/brownout circuit, off-chip 62-KB FLASH ROM and 2 × 128-KB RAM banks (XRAM), plus a 16-KB non-volatile memory chip (EEPROM) for storing system configuration information. A 25 MHz CPU clock speed was employed in the design. Although the C167 has on-chip ADC converters, the resolution was limited to 10-bits. To increase the resolution to a more acceptable level, an 8-channel 14-bit self-calibrating ADC converter (the AD7856 from Analog Devices Ltd.) was employed. An SPI interface was employed to the host CPU, and 5 V Zener diodes were employed to prevent the ADC input circuitry being damaged by any excessive voltages. The hardware employed in the system is shown schematically in Figure 4. Sampling of input channels occurred at 100 Hz frequency, using a PWM signal generated by the C167 hardware. Very little host CPU management was therefore required; hence, sampling jitter was reduced. The main intervention required by the host CPU was for the extraction of the conversion values once every 10 ms, a jitter-insensitive task.

### 3.2. Software Architecture

**Figure 5:**
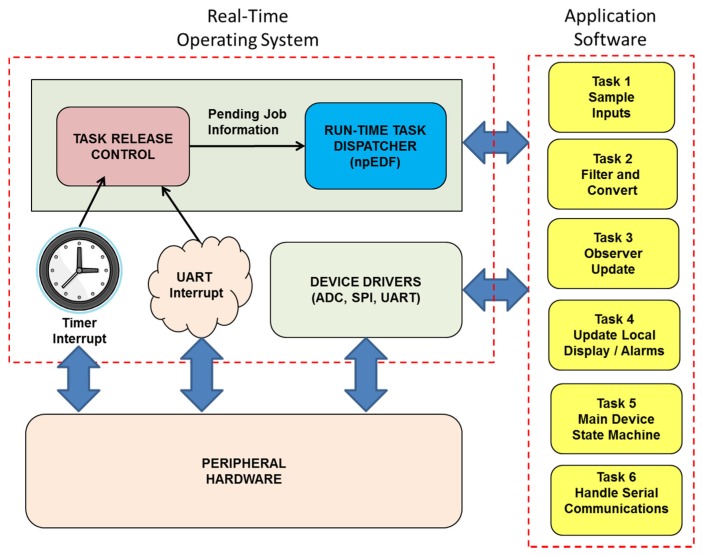
Main software elements employed in the system.

Upon power on/reset, the system first performed a software-based self-test, consisting of a ROM checksum test, a RAM ‘March’ test followed by a timer/oscillator self-test. The latter test compares the amount of CPU cycles elapsing over a fixed duration of the on-chip hardware timers, and compares these values with the time taken to charge an external resistor/capacitor network with fixed time constant. If these tests are passed successfully, a system initialization function is called. In addition to performing system initialization tasks such as setting up the ADC converter, the nine tunable parameters that define the observer constants are extracted from an EEPROM. Following this, a task scheduler is started. The application software functionality was broken down into six separate tasks. The run-time software architecture was as shown in Figure 5.

The first task was required to extract the five ADC channel conversion readings from the AD7856 via an SPI link to obtain the input signals. The second task was then required to filter the signals using recursive first-order low pass filters, and convert these readings into appropriate engineering units. The third task evaluates the observer models, implementing equations (1–4). Based on the filtered outputs of the observer, the fourth task drives the local indicator status LED’s. To allow the observer to converge upon power on/reset, the software is required to suppress any faulty/degrading outputs for a user-adjustable period, typically no more than 10 s. The fifth task updates the main system state machine once every 50 ms. The sixth and final task handles the Modbus Slave communications via the hardware UART (Universal Asynchronous Receiver/Transmitter), which handles buffered character reception and transmission over the 57,600 bps serial link. Static checking was routinely employed to help enforce best-practice coding techniques during development of the application software, which also included application of the MISRA rules and related guidelines [12,13,14].

Although the dynamics of the monitored system are relatively slow moving, the efficiency of the observer is related (in part) to the selected sampling rate, as the closer the observer approximates a continuous function the better the performance will be [9]. For this reason, as described above the selected sampling rate was 100 Hz, with cut-off frequencies for the input digital filters selected as 10 Hz. The local communications were updated every 100 ms. In order to meet the real-time constraints of the application, a non-preemptive Earliest Deadline First (npEDF) task scheduler was employed to control the timing of task executions. This was because of the very low overheads it requires (specifically, very low RAM/ROM and CPU overheads, with no shared resource handling protocol needed), coupled with–amongst other things-its optimality among the non-idling, non-preemptive schedulers and its suitability for use with both periodic and sporadic tasks [15]. In such a system, however, task execution times are required to be comparatively short to prevent undue blocking occurring. Tasks one to five were created as periodic tasks, with the required periods. As waiting for the UART transmit or receive register to be available, task six (the serial link update task) was created as a sporadic multi-stage task [12], with transmission and reception buffering employed along with the 16-byte transmit/receive FIFO on the C167 hardware. The software was written in embedded ‘C’, and compiled/linked with the Keil xC16x development toolchain. Timing was verified using the sufficient schedulability condition based on CPU utilization for npEDF derived previously [16]. Further descriptions of the verification of the functional and timing properties of the software for the prototype edge device have been documented in an earlier work [17].

### 3.3. Communications Architecture

**Figure 6:**
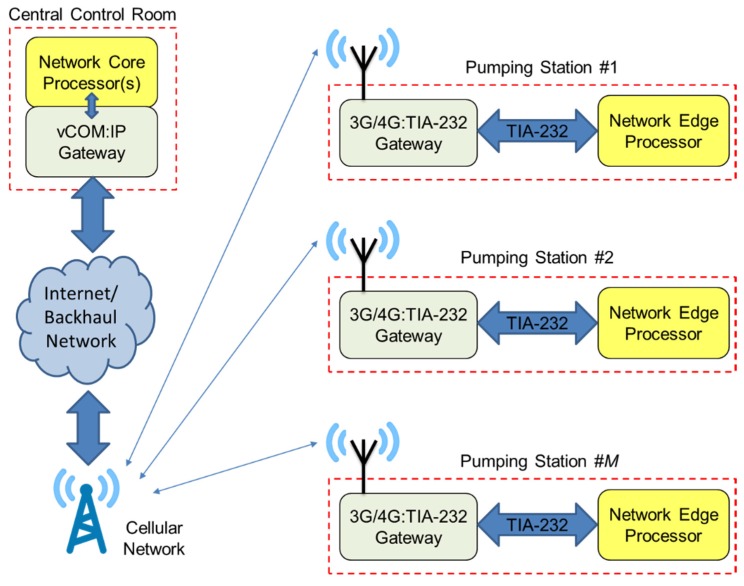
Overall communications architecture of the proposed system.

An overview of the communications architecture of the proposed system is shown schematically in Figure 6. As mentioned, the device software and hardware configuration employs a 56,700 bps TIA-232 Serial link for communications. The device supports the Modbus Supervisory Control and Data Acquisition (SCADA) protocol, and implements a Modbus RTU slave in software. When valid Modbus requests arrive, a software task handles the requests and prepares the required responses. A cellular Serial/GSM gateway provided (remote) IP connectivity to allow integration into the IoT. The central control station implements a Modbus RTU master implemented in software, with periodic/sporadic Modbus transmissions and retransmissions to slaves handled using existing master-slave scheduling techniques [18]. A virtual COM port (vCOM) driver provides a gateway to carry the Modbus RTU frames over Internet Protocol and across the Cellular network. In the prototype design, the OnCell G3150A-LTE Serial Gateway from Moxa^®^ was employed to provide cellular IP connectivity in the prototype Edge device, with end-to-end 256-bit encryption enabled. This provided end-to-end secure duplex serial communications to carry the Modbus traffic between the network Core and the Edge device. In future developments, flexible scheduling of Core-Edge communications and off-loading of analytics workload between the Core and Edges will be implemented using state-of-the-art techniques, such as those described in [19].

## 4. Offline Validation Testing

**Figure 7:**
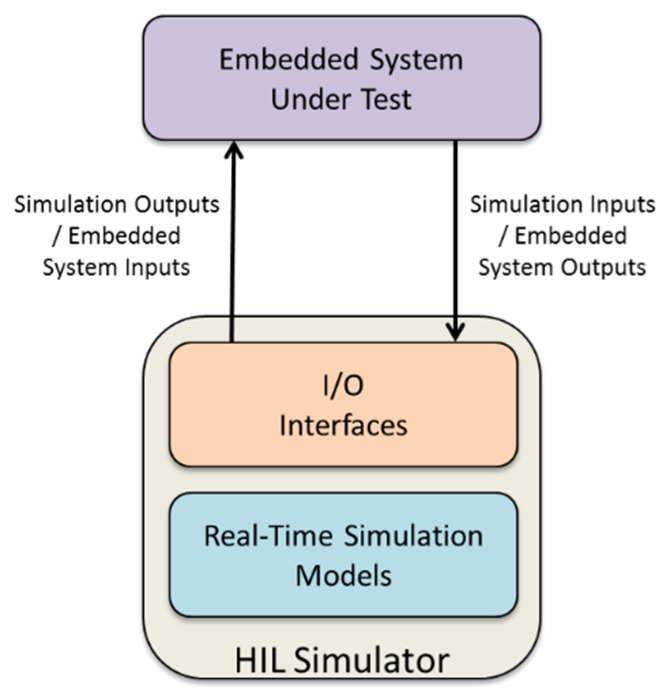
Overview of Hardware-In-The-Loop (HIL) testing of embedded real-time systems.

Hardware-In-The-Loop (HIL) testing as a means for both early and late-stage system validation in real-time and embedded systems is now a well-established concept [12,20,21]. The principle of HIL simulation of an embedded system is illustrated in Figure 7; the unit under test (UUT) executes on representative hardware, and its outputs are fed directly to the simulator. The UUT outputs are sampled and used as input variables to a host dynamic simulation model, which is evaluated in real-time. The simulation outputs, which are synthesized representations of signals internal to the dynamic model, are then fed back to the UUT thereby closing the control loop. Since the UUT is representative of the final system implementation, many types of specification defect, omissions or otherwise unexpected interactions that may result in failures or otherwise unacceptable behaviors may be removed before the system enters operational service. However, the simulation must be designed and implemented correctly in order for results to be reliable. If this can be achieved, then HIL testing can be regarded as a representative virtualization technique which allows a potentially large design space to be explored and tested, with few (if any) consequences should the UUT malfunction or behave inappropriately at run-time. 

**Figure 8:**
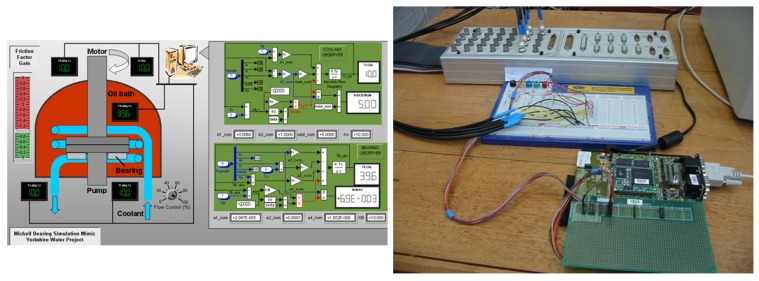
HIL testing of the prototype edge device on a C167 processor.

As mentioned, during development of the observer equations, detailed models of the pumping process were created for validation purposes. The popular design and analysis package Simulink© was employed for their implementation. In order to allow HIL testing of the edge device to take place, the Simulink models of the process were employed along with real-time model simulation and interface devices provided by dSpace©. This allowed three scenario’s (baseline, coolant flow fault and bearing friction fault) to be validated on the embedded implementation. A screenshot of the HIL user interface and testing procedure is as shown in Figure 8. In addition to simulation of the bearing unit itself, type *k* thermocouples along with signal transmitters and signal isolators of the same characteristics as employed in the field trials (to be described in the following Section) were simulated as a part of the overall model.

**Figure 9:**
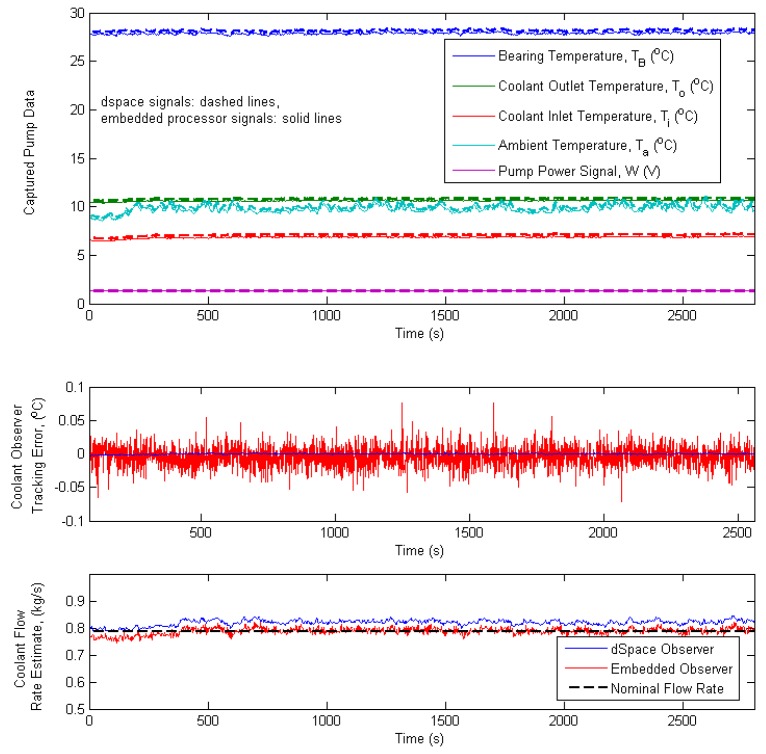
Comparative analysis of the Simulink-based and edge device-based observers (baseline case).

**Figure 10:**
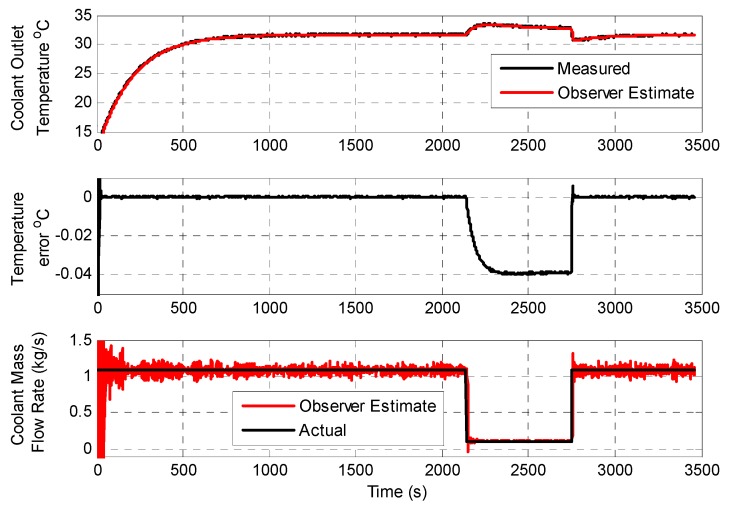
Comparative analysis of the Simulink-based and edge device-based (coolant flow fault case).

**Figure 11:**
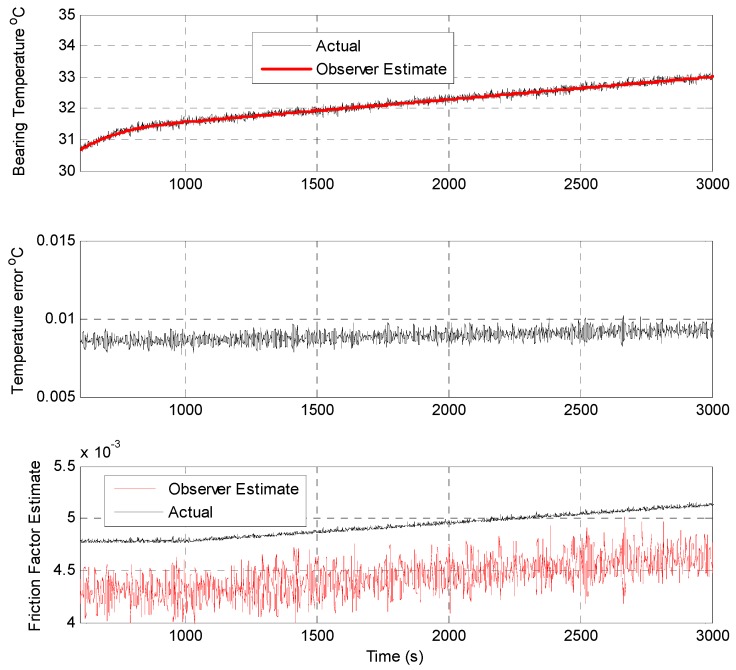
Comparative analysis of the Simulink-based and edge device-based observers (bearing friction factor fault case).

Figure 9 shows data captured comparing the Simulink-based observer implementation with the edge device observer implementation during the baseline scenario. Figure 10 shows data captured comparing the Simulink-based observer implementation with the embedded observer implementation during the coolant flow fault scenario. In this fault scenario, the coolant flow rate is dropped significantly 2150 s into the simulation run, and remains as such for a duration of 600 s. As can be seen in the captured results, although the coolant flow is not a directly instrumented parameter, the observer implementation accurately tracks the coolant temperature and estimates the reduced coolant flow condition. Figure 11 shows data captured comparing the Simulink-based observer implementation with the embedded observer implementation during the coolant flow fault scenario. In this fault scenario, the bearing friction factor *μ* is allowed to drift upwards starting 1000 s into the simulation run, and increases slowly over time thereafter. As can be seen in the captured results, although the bearing friction factor *μ* is a non–measurable model parameter, the observer implementation detects and starts to track the gradual increase in the parameter, albeit with a small offset.

In all tested scenarios, the results obtained indicated that the observer implementations were accurate enough for the intended purpose, giving results almost indistinguishable from the (known) internal variables in the Simulink model–with less than 5% maximum and 1% average (mean) errors recorded for the estimated quantities. As such, testing progressed to field trials.

## 5. Online Validation Testing

**Figure 12:**
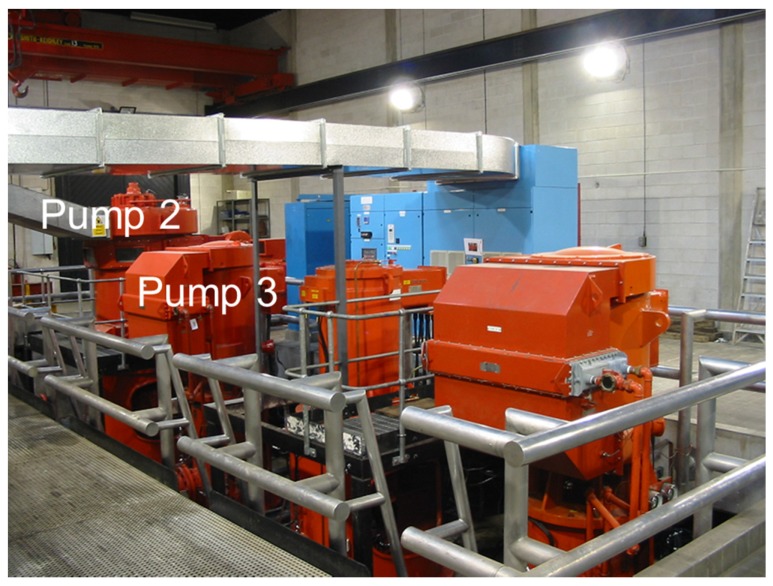
Arrangement of pumps inside pumping house, showing pump 2 (**left**), pump 3 (**middle**), and pump 1 (**right**). Pipework is housed out of view under the metallic walkway.

Following the successful off-line validation testing, implementation tests of the edge device were then performed on Sulzer© Water Pumps at Lobwood pumping station, West Yorkshire, UK. As shown in Figure 12, the pump house contains three pumps; two of which are active at any point in time, with the other being ready as standby in case failure. The active pumps are rotated on a scheduled basis, allowing routine checks and maintenance to be carried out on the third. The use of the observer implementation to detect bearing wear and coolant flow failure will clearly provide guidance allowing optimization of the maintenance schedule, helping to prevent both active pump failures and unnecessary changeover and maintenance. During online testing, the observer implementation was deployed on pumps two and three as indicated in Figure 12.

**Figure 13:**
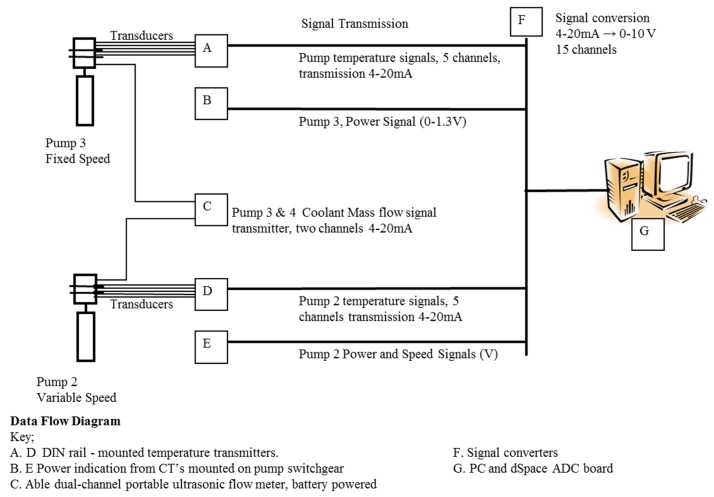
I/O Schedule for online testing of the device implementation.

**Figure 14:**
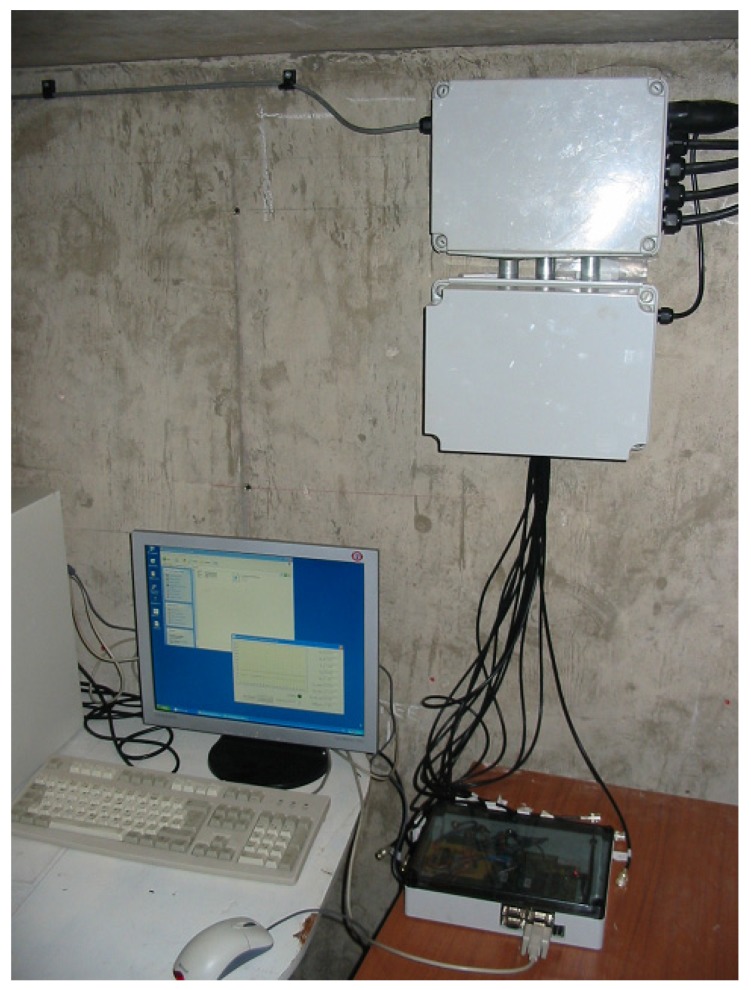
Device implementation in IP67 enclosure (bottom right), interface to local PC (bottom left) and instrumentation/communication interfaces (top right).

The Input/Output schedule for the observer implementation was as shown in Figure 13. Temperatures were measured via externally mounted, low-cost type-*k* thermocouples. ALM-46 low-cost head-mount transmitters were employed to convert effective signal ranges to current transmission, and COM-3B signal isolators were employed to convert to current to voltage prior to conversion by the ADCs. The calibration of the combined sensor, transmitter, isolator and ADC for each temperature measurement channel was as follows: [0 °C:100 °C] → [4 mA:20 mA] → [0 V DC:5 V DC] → [0000*_h_*:0x3FFF*_h_*]. Stated combined accuracies of the transmitter and isolator (due to linearization) were ±0.1% and ±0.15% of full-scale max, and the combined accuracy of the ADC converter was ±0.0015% max. Although a full meteorological analysis is deferred to future work, the accuracy of these measurement chains seems adequate to reproduce results of similar accuracy in the prototype field trials as in the laboratory-based HIL test results, as models of the same thermocouples, signal transmitter and signal isolator were employed in the laboratory simulations. During testing, a portable ultrasonic flow meter was used to measure coolant mass flow rates and hence help to calibrate the observer. Where appropriate, information on pump speed and power was obtained directly from the electronic switchgear. The observer device was mounted in an IP67 enclosure with input and output connections. The observer was installed on-site for long-term trials with data recorded to a standard desktop via the TIA-232 point-to-point connection, as shown in Figure 14. In total, neglecting wiring costs, component costs for the encapsulated observer implementation were ≈ £200.

**Figure 15:**
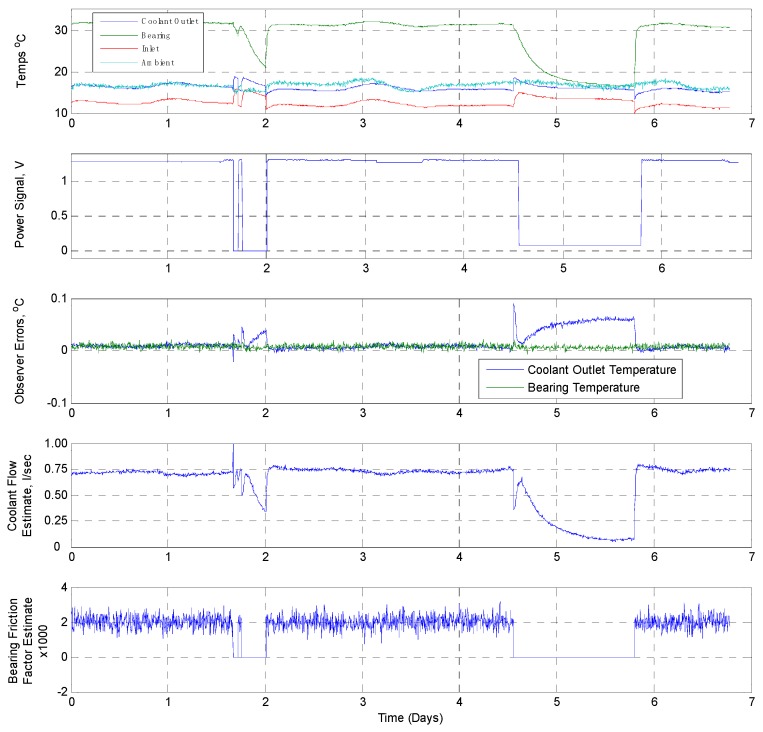
Online edge device implementation site trials results data: Pump 3 (fixed speed).

As shown in Figure 15, the online site trial for Pump 3 (fixed speed) took place over a period of 7 days in which the CM system ran uninterrupted. Tracking of the observer was highly effective, with the estimated coolant flow remaining constant except during shut down periods (day 2 and day 4/5), when the flow rate fell to zero following the bearing temperature cooling (as expected). The bearing friction factor remained constant at the expected level, again indicating fault free operation, aside from during the shutdown periods when it fell to zero (as expected).

**Figure 16:**
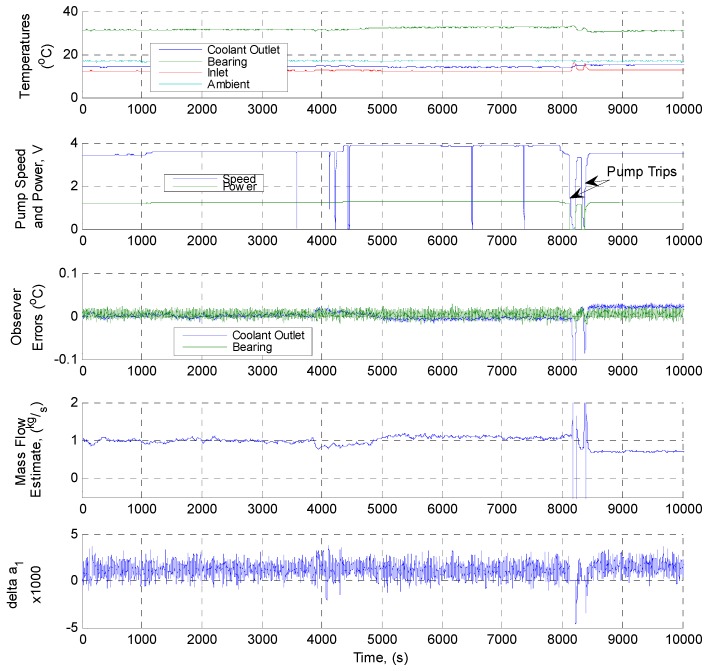
Online edge device implementation site trials results data: Pump 2 (variable speed).

Figure 16 shows the online site trial for Pump 2 (variable speed) which again took place over a period of 7 days in which the CM system ran uninterrupted. Tracking of the observer was again just as effective, with the estimated coolant flow remaining piecewise constant, tracking small speed changes at several points during the trial. During the course of the experiment, pump trips occurred eight times; during the longer of these trip events (occurring 8000 s into the trial) the estimated flow rate fell to zero following the bearing temperature cooling (as expected). The bearing friction factor remained constant, again indicating fault free operation independent from pump load, aside from during the longer trip event when it fell considerably (as expected). In summary, both the on-line and off-line simulations and tests demonstrated that the observer-based edge device functioned as expected, and that parameter estimations were within acceptable limits for the proposed application. During the course of the field trials, however, it was noted that some simple improvements to fault detection and annunciation logic will be required for some specific situations when progressing from trials to continuous operation; most notably, supressing fault detection and annunciation during shut-down and trip conditions. These, and other improvements, are currently being implemented, and a full metrological analysis of the modified system is underway. Overall, however, as the device is a relatively low-cost solution (as mentioned approximately £200, excluding cabling, gateway, and network access costs) in comparison with the value of the plant, this gives a very promising indication of its overall suitability.

## 6. Conclusions and Further Work

In this paper, developments towards digitization in the water industry have been presented. Specifically, the development, validation, and field-testing of a real-time edge device as part of a CM/PM system for deployment upon large-scale pumping equipment in the water industry has been described. Initial field trials with the observer-based edge device have been very promising. In addition, simulation results and accuracy analysis indicate the system is fit-for-purpose. Future work will describe longer-term experiences and analyze the fault-detection capability of the proposed embedded solution. Future work will also describe developments related to integration of multiple CM edge devices to the network core, and progress the analytics required for optimized operation and PM of the pumping equipment within a wider Industry 4.0 framework.
